# Inbreeding Effects on Quantitative Traits in Random Mating and Selected Populations of the Mulberry Silkworm, *Bombyx mori*

**DOI:** 10.1673/031.012.14001

**Published:** 2012-12-05

**Authors:** Jamuna Doreswamy, Subramanya Gopal

**Affiliations:** Department of Studies in Sericulture Science, University of Mysore, Manasagangotri, Mysore 570 006 Karnataka, India

**Keywords:** breeding, germplasm centre, REML, residual maximum likelihood method

## Abstract

The objective of the present study was to estimate the level of inbreeding coefficient during inbreeding of the pedigree of random mating and selected populations of two distinct races of mulberry silkworm, *Bombyx mori* (Lepidoptera: Bombycidae), in the silkworm germplasm. The six generation data of the two races, namely multivoltine *Pure Mysore* and bivoltine *NB_4_D_2_*, were studied for inbreeding depression coefficient using the residual maximum likelihood method, utilizing two statistical models by analyzing six quantitative traits, namely, larval weight, cocoon weight, shell weight, shell ratio, pupation rate, and filament length. The results of the present experiment demonstrated that the inbreeding coefficient was significant in Model 1 for most of the economic traits in the random mating populations of both the races compared to those of selected populations. These results suggest that during stock maintenance, application of rigid selection for increased numbers of generations helps to retain original characteristics of the pure races while reducing the deleterious effects of inbreeding. The significance of inbreeding coefficient is discussed with reference to the inbreeding of silk moths in the silkworm germplasm.

## Introduction

The mulberry silkworm, *Bombyx mori* L (Lepidoptera: Bombycidae), is a lepidopteron insect known for the production of silk (“queen of natural fibers”), and extensive genetic studies have been carried out utilizing this insect (Tazima 1978). Sericulture, an important source of revenue generation in many countries, is based on the maintenance of pure races belonging to different voltinistic groups in germplasm centers. Sib-mating of the progenies derived from a single brood is preferred, and original traits of the silkworm races are protected to a great extent. The homozygous races maintained in the germplasm centers are subsequently crossed to produce hybrids for commercial exploitation. As a result, silkworm breeders retain the original characters of the races through selection pressure to avoid inbreeding depression.

The inbreeding depression for a trait is a linear function of the inbreeding coefficient (Lynch and Walsh 1998). One of the measures of inbreeding is the inbreeding depression coefficient, which corresponds to half of the relation between parents. Utilization of the linear-regression method to estimate inbreeding depression for production traits in livestock breeding is well documented (Allaire and Henderson 1965; Henderson 1984; Hudson and Van Vleck 1984; Wall et al. 2005). In sand cricket, *Gryllus firmus*, it is reported that inbreeding depression can cause a substantial decrease in trait values, and life history traits show significantly higher inbreeding depression than morphological traits (Roff 1998). In mulberry silkworm, there are a few reports on the inbreeding coefficient for economic traits of cocoons (Nematollahian et al. 2010; Azizian et al. 2011). The problem of applying inbreeding to silkworm population has been discussed by Petkov et al. (1999). However, there is no information available in regard to the effects of selection on inbreeding depression coefficient for various traits in germplasm centers. Thus, the purpose of this research was to study (1) the level of inbreeding depression coefficient for six quantitative traits in two races inbred for six generations and (2) the differential expression of inbreeding coefficient for two different models.

## Materials and Methods

In order to estimate the level of inbreeding and investigate the occurrence of inbreeding depression through inbreeding coefficient in the random mating (non-selected) and selected populations, a pilot study was undertaken by drawing two distinct races of the mulberry silkworm, namely multivoltine Pure Mysore and bivoltine NB_4_D_2_, from the germplasm bank of the Department of Studies in Sericulture Science, Mysore, India. The parental population of the two races were grouped into random mating and selected populations. In the random mating populations, selection was not applied for the six traits under study, whereas for the selected populations, selection pressure was applied at every stage of the life cycle of silk moth (egg, larva, pupa, and adult) comprising six quantitative traits (larval weight, cocoon weight, shell weight, shell percentage, pupation rate, and filament length), which were selected during rearing of silkworm for six consecutive generations. The two populations were maintained in replicates of three for statistical analysis. The results were computed according to the equation described by Falconer (1989), and the single trait linear equation was applied as detailed below.

Rate of Inbreeding per generation: F_t_= ΔF + (1-ΔF) F_t-1_ Where: F_t_ = inbreeding at generation t, F_t-1_ = inbreeding at generation t-1, ΔF = inbreeding coefficient.

The next step is the estimation of the ΔF from the above formula. The rate of inbreeding (ΔF) was estimated as the difference between the inbreeding of the individual (F_t_) and the average inbreeding of the parents (F_t-1_) divided by (1- F_t-1_). (Falconer and Mackay 1996).


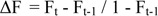


When the inbreeding coefficient is expressed in terms of ΔF, the above formula is valid for any breeding system and is not restricted to the idealized population.

For repeated full sib-mating, the inbreeding coefficient settles down to a constant value of 0.191/generation. Hence, ΔF = 0.191/generation (Falconer 1989). Based on the above equation, the data is subjected to two linear mixed-models, using the residual maximum likelihood method directive in GENSTAT 9 (2006). In Model 1, each quantitative trait was included as one response variable and fixed effect, which is a covariate and includes interaction between races, replicates generation, and is a constant value. In Model 2, in addition to the above, generation squared (G^2^) was added to the fixed effects.

Model 1 = constant + races + races^*^ replicates + ΔF + generations

Model 2 = constant + races + races^*^ replicates + ΔF + generations + generations^2^

The formula is as follows:
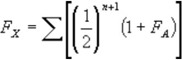

Where F_x_ is the coefficient of inbreeding of individual x, n is the number of connecting links between the two parents of x through common ancestors, and F_A_ is the coefficient of inbreeding of the common ancestor.

## Results and Discussion

The mean values and inbreeding coefficient (ΔF values) for the two races, both in the random mating and selected populations of silkworm, are listed in Table 1, and the results are herein discussed.

**Table 1.**
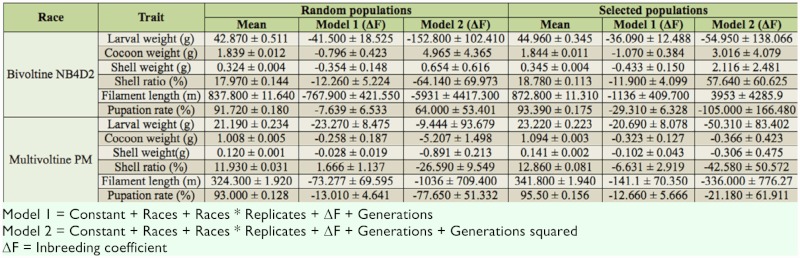
Inbreeding coefficient for six quantitative traits in the random mating and selected populations of the two races of the mulberry silkworm, *Bombyx mori*.

### Random mating populations

In Model 1, all six analyzed traits in the random populations exhibited negative significant ΔF values where generation (G) was fitted as one of the covariates. In the bivoltine NB_4_D_2_ race, the least ΔF value being observed for the trait shell weight (- 0.354) and the highest for filament length (- 767.9) where as in multivoltine Pure Mysore race the ΔF value ranges from a lowest of -0.028 for shell weight to a highest of -73.277 for the trait filament length.

In Model 2, where G^2^ was fitted as one of the covariates, two of the six traits, namely larval weight and filament length, analayzed in the NB_4_D_2_ race showed significant negative ΔF values (-152.800 ± 102.410 for larval weight and -5931 ± 4417.3 for filament length), and a non-significant negative ΔF value was observed only for one trait, namely shell ratio (-64.140 ± 69.973). Contrary to the above, the other three traits, namely cocoon weight (4.965 ± 4.365), shell weight (0.654 ± 0.616), and pupation rate (64.000 ± 53.401), exhibited respective positive significant ΔF values. In the case of the multivoltine Pure Mysore race, five of the six traits fell into the negative significant group, while larval weight fell into the negative non-significant group (-9.444 ± 93.679). The lowest ΔF values appeared for cocoon weight and shell weight in both the races. In the progenies of the random mating populations, the low inbreeding depression coefficient values (e.g., cocoon weight and shell weight) in both the models clearly indicate low dominance variance for these traits, and vice versa is true for progenies of selected populations (e.g., filament length).

### Selected populations

In the selected populations of the two races studied in Model 1, all six traits showed negative significant ΔF values. In the bivoltine NB_4_D_2_ race, the lowest and highest ΔF values were -0.433 for shell weight and -1136 for filament length, respectively. Similarly in the Pure Mysore race, the lowest ΔF value (-0.102) was recorded for shell weight, and the highest ΔF value (-141.1) was recorded for filament length. The traits cocoon weight and shell weight exhibited low ΔF values in both the races under study.

In Model 2, all six traits selected for analysis recorded non-significant ΔF values for both the races. In the NB_4_D_2_ race, larval weight (-54.950 ± 138.066) and pupation rate (-105.000 ± 166.480) showed negative non significant ΔF values, whereas the remaining four traits (cocoon weight, shell weight, shell ratio, and filament length) exhibited respective positive significant ΔF values of 3.016 ± 4.079, 2.116 ± 2.481, 57.640 ± 60.625, and 3953 ± 4285.9. The lowest value noticed was for shell weight (2.116), and the highest value noticed was for filament length (3953). In the Pure Mysore race, all the traits showed negative non-significant ΔF values. The lowest value was recorded for shell weight (-0.306) and followed by cocoon weight (-0.366). The highest value was for filament length (-336.0). Cocoon weight and shell weight had the lowest ΔF values in both the races. It is quite interesting to note that both the lowest (shell weight) and highest (filament length) inbreeding depression coefficient values were noticed for the same traits in random mating and selected populations of the two races. From the results it is apparent that when Model 1 was applied, irrespective of the type of the population and the involvement of selection pressure, all the traits showed significant inbreeding depression. However, when Model 2 was applied, the results clearly demonstrated that in random mating populations most of the traits showed significant inbreeding depression values, except shell ratio in bivoltine NB_4_D_2_ race and larval weight in Pure Mysore race. In selected populations for the same model, all the traits being studied revealed non-significant inbreeding depression values. The absence of inbreeding depression in selected populations for all the traits studied is an indicative of the dominant gene effect.

The analysis of the results by applying the two models clearly indicates that during stock maintenance in germplasm centers, application of rigid selection for more numbers of generations helps in the retention of original characteristics of the pure races and also reduces the deleterious effects of inbreeding. Thus, the net effort of inbreeding in the selection program will depend on the magnitude of the selection response relative to the possible depression and rate of accumulation of inbreeding, as was reported by Swanepoel et al. (2007) in sheep. A comparative account of the values of ΔF between the two populations being studied clearly revealed low ΔF values for cocoon weight and shell weight, two traits that are important to silk production. Therefore, if variability is not introduced through selection, there are possibilities of increases in inbreeding coefficient values, and the deterioration of these traits are expected.

In conclusion, it is suggested that the breeders of germplasm take notice of ΔF values. Prolonged maintenance of stocks (G^2^) with rigid selection, rather than short duration (only G) of selection, will be useful in race maintenance programs to minimize the inbreeding depression.
